# A Common Dataset for Genomic Analysis of Livestock Populations

**DOI:** 10.1534/g3.111.001453

**Published:** 2012-04-01

**Authors:** Matthew A. Cleveland, John M. Hickey, Selma Forni

**Affiliations:** * Genus plc, Hendersonville, Tennessee 37075; † School of Environmental and Rural Science, University of New England, Armidale NSW 2351, Australia

**Keywords:** pig, genomic relationships, GenPred, cross-validation, shared data resources

## Abstract

Although common datasets are an important resource for the scientific community and can be used to address important questions, genomic datasets of a meaningful size have not generally been available in livestock species. We describe a pig dataset that PIC (a Genus company) has made available for comparing genomic prediction methods. We also describe genomic evaluation of the data using methods that PIC considers best practice for predicting and validating genomic breeding values, and we discuss the impact of data structure on accuracy. The dataset contains 3534 individuals with high-density genotypes, phenotypes, and estimated breeding values for five traits. Genomic breeding values were calculated using BayesB, with phenotypes and de-regressed breeding values, and using a single-step genomic BLUP approach that combines information from genotyped and un-genotyped animals. The genomic breeding value accuracy increased with increased trait heritability and with increased relationship between training and validation. In nearly all cases, BayesB using de-regressed breeding values outperformed the other approaches, but the single-step evaluation performed only slightly worse. This dataset was useful for comparing methods for genomic prediction using real data. Our results indicate that validation approaches accounting for relatedness between populations can correct for potential overestimation of genomic breeding value accuracies, with implications for genotyping strategies to carry out genomic selection programs.

The availability of common datasets is important to the scientific community. These datasets can be used to compare and benchmark methods, to provide links between different datasets facilitating meta-analysis, and to enable researchers who lack such resources to test hypotheses that address important questions. In human genetics, many common datasets are available, *e.g.* International HapMap Project (The International HapMap Consortium 2003), 1000 Genomes (The 1000 Genomes Project Consortium 2010), Framingham Heart Study (Dawber *et al.* 1951), Mouse Genomes Project (Keane *et al.* 2011), and have led to numerous discoveries.

Because commercial data may hold economic value, common datasets of meaningful size have in general been unavailable in livestock, particularly during the genomics era in which organizations have made large investments in genotypes and phenotypes. Efforts to implement genomic selection in a number of livestock species have generated datasets that contain multigenerational phenotypes, genotypes, pedigree, and progeny test-derived estimated breeding values (EBV). Such commonly available datasets could be used in numerous ways, in particular to evaluate new methods to estimate genomic breeding values (gEBV). Additionally, important questions relating to the nature and properties of genomic selection (GS) could be assessed with such a dataset, including the effect on inbreeding (Daetwyler *et al.* 2007), the importance of close relatives when training prediction equations (Habier *et al.* 2010; Clark *et al.* 2011), and the dynamics of long-term response to selection (Jannink 2010).

As part of the effort to implement GS in pigs (*Sus scrofa*), PIC (a Genus company) created several datasets containing individuals with phenotypes for a number of traits, high-density genotypes (60k), polygenic estimated breeding values (pEBV; no genomic information) for pure and crossbred traits, and complete pedigree. PIC has implemented several methods for routine genomic evaluation, including a two-step approach using BayesA/BayesB (Meuwissen *et al.* 2001) and the single-step evaluation described by Misztal *et al.* (2009), which, based on extensive internal testing, PIC considers current best practice for predicting gEBV in a practical setting. Genomic breeding values from these methods can be routinely predicted through a process that includes quality checks and data editing, imputation of missing genotypes, and validation in “selection candidates” when pEBV accuracies approach progeny test proofs. These validations can be problematic in pig populations in which the structure of the genetic improvement program yields few individuals with high accuracy pEBV across traits. Thus, we require optimal validation procedures that avoid overestimating the expected accuracy of the gEBV.

This article describes a pig dataset that PIC has made available to the scientific community for testing and validating alternative methods for genomic prediction (File S1). In addition, current best-practice methods were applied to the data to predict and validate gEBV. We discuss peculiarities particular to the data structure, with an emphasis on their impact on gEBV accuracy from validation.

## Materials and Methods

### Data

The dataset consisted of 3534 animals from a single PIC nucleus pig line with genotypes from the Illumina PorcineSNP60 chip (Ramos *et al.* 2009) and a pedigree including parents and grandparents of the genotyped animals (N = 6473). The majority of genotyped animals were selected for the genomic evaluation of a specific trait, and the remaining were added as part of a strategy to “fill-in” missing herd sires and sows to calculate genomic breeding values for selection candidates. The sample consisted of male and female pigs born since 2000, with varying pedigree relationships among animals, although the original selection avoided sampling multiple members of full-sib families.

#### Phenotypes:

**Figure 1:**
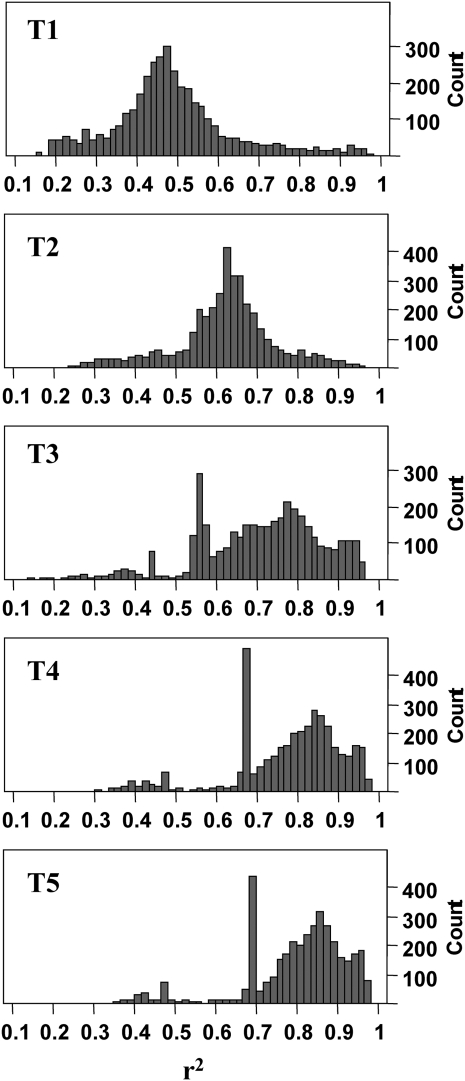
Accuracy (r^2^) of estimated breeding values for genotyped animals (N = 3534).

Genotyped animals had phenotypes for five purebred traits (phenotypes in a single nucleus line), with heritability ranging from 0.07 to 0.62 (Table 1), which represent a small number of phenotypes that are routinely collected from birth in the genetic nucleus. Each phenotype was either corrected for environmental factors (*e.g.* year of birth or farm) and rescaled by correcting for the overall mean (traits 3, 4, and 5) or was a rescaled, weighted mean of corrected progeny phenotypes (traits 1 and 2), for which many animals have no individual performance data. Each genotyped animal also had pEBV and accuracies from single-trait pedigree-based BLUP evaluations. The models to calculate corrected phenotypes and the models to predict pEBV included the full PIC pedigree and all data used in a typical production run for each trait, excluding any genomic information. Accuracy was of the form $1\;-\;\frac{PEV}{\sigma_{A}^{2}}$, where PEV is the prediction error variance and $\sigma_{A}^{2}$ is the additive genetic variance. The distribution of accuracy for all traits is depicted in Figure 1. Animals were originally selected to maximize the pEBV accuracy of trait 2.

**Table 1:** Summary of phenotype data

Trait	N	Mean	SD	h^2^^*a*^	Var(a)^*a*^
T1	2804	−0.045	1.21	0.07	0.22
T2	2715	0.005	1.12	0.16	2.11
T3	3141	0.706	0.96	0.38	0.66
T4	3152	−1.073	2.33	0.58	4.93
T5	3184	37.989	60.45	0.62	3459.09

h^2^, heritability; var(a), additive variance.

a Heritability and additive variance estimated from the full PIC production dataset.

#### Genotypes:

Genotypes available from the PorcineSNP60 chip (N = 64,233) were filtered for extreme minor allele frequency (<0.001) and proportion missing genotypes by SNP (>10%). Additionally, markers on the X or Y chromosome were excluded, yielding 52,842 total SNP. The overall missing genotype rate was less than 1%, but many GS methods require complete nonmissing genotypes. AlphaImpute (Hickey *et al.* 2011) was used to calculate probabilities of each parental allele, which are combined to fill in any missing genotypes. An imputed genotype based on a probability score [the sum of the allele probabilities (SAP) yields a non-integer genotype ranging from 0 to 2] was used to replace missing information. SNP with both known and unknown position were included and imputed, but the map order was randomized and SNP identity was recoded.

#### Genotyping strategy:

The original selection of animals for genotyping aimed to minimize pedigree connections between selected individuals, but a high level of relatedness remained due to the breeding schemes used in genetic nucleus pig lines (Table 2). The populations in the nucleus are relatively small by line, and the turnover in breeding animals can be rapid, which makes it difficult to identify animals with high-accuracy breeding values to construct a training set. The animals selected for genotyping tend to be used widely and have more close relatives than others in the population. One advantage of applying GS in pig populations, though, is the high levels of relatedness between genotyped and phenotyped training animals and genotyped selection candidates. The gEBV accuracy in selection candidates can therefore be maintained through incremental increases to the existing training dataset by adding small numbers of herd boars (and potentially sows) to sustain connections across generations. This structure, however, makes validation of SNP effects in a less-related population problematic, and thus inferences about the usefulness of alternative methods in other livestock species may be difficult.

**Table 2:** Percentage of genotyped individuals with relatives genotyped in dataset, by category of relative

Genotyped Relatives Category	% Genotyped Relatives
Sire	62
Dam	40
Sire + Dam	29
PGS + PGD	24
MGS + MGD	21
PGS + PGD + MGS + MGD	8
Offspring	34
Offspring + Ancestor*a*	30

PGD, paternal grand-dam; PGS, paternal grand-sire; MGD, maternal grand-dam; MGS, maternal grand-sire.

a At least one offspring and at least one ancestor are genotyped.

### Genomic breeding value prediction

Prediction of gEBV in PIC has taken two forms since the development of and subsequent large-scale genotyping on the PorcineSNP60 chip. Breeding animals in major lines have been routinely genotyped for 60k SNP and used as training populations to estimate SNP effects and to identify important markers for traits of interest using de-regressed breeding values and Bayesian analysis approaches [*e.g.* GenSel; Fernando and Garrick (2009)]. Based on results from training populations, a large number of selection candidates have been genotyped for smaller trait-specific panels. The resulting gEBV have then been blended with the polygenic breeding value, using an approach similar to VanRaden *et al.* (2009), and incorporated into the overall index for selection. Alternatively, the single-step approach of Misztal *et al.* (2009) has been implemented to reduce the computational and logistical requirements of the multistage Bayesian approaches. This method uses all available SNP to construct a genomic relationship matrix among genotyped individuals, which is then combined with the standard numerator relationship matrix that includes all un-genotyped individuals. Genomic breeding values are then predicted for all animals, regardless of genotyping status. In this situation, selection candidates can be genotyped for a small panel (*e.g.* <1,000 SNP) and 60k genotypes imputed to improve the predictive power.

To predict gEBV using these general approaches, the data were further filtered to exclude genotypes with extreme minor allele frequency (<0.02) and large values for the Pearson chi-squared test statistic (>300), indicating extreme deviation from the expected genotype proportions. A total of 48,866 SNP remained for analysis. To evaluate the accuracy of methods to predict gEBV, a 6-fold cross-validation procedure was used (XVal), in which all animals appear in the training set and in the validation set. Animals were randomly assigned an integer from the set (1,6) for assignment to a fold. Each analysis was performed to predict gEBV for the validation set using all remaining individuals as the training set. This was repeated so that each animal was in the validation set one time and in the training set five times. The training set can be defined as animals with phenotypes and high-density genotypes, whereas animals in the validation set have only high-density genotypes. Each training set, therefore, consisted of 2945 genotyped animals (reduced to the number of animals with phenotypes in that set), with the remaining in validation where phenotypes were removed. Genomic breeding values were estimated using BayesB (as implemented in GenSel) on both phenotype (BayesB_ph) and de-regressed pEBV (BayesB_ebv) of genotyped animals and using phenotypes in the single-step approach (SStep). Additionally, a standard BLUP using phenotypes but no genomic information was performed (BLUP). EBV and gEBV were calculated for all genotyped animals.

### Genomic breeding value validation

Accuracy was estimated as the correlation between gEBV and high accuracy pEBV, which is a substitute for the true breeding value. In this case, many animals did not have accuracies that could be considered high (*e.g.* >0.90); therefore, a subset of each validation set was selected based on pEBV accuracy. The top 75 animals by pEBV accuracy for each trait and cross-validation were selected (N = 450) to correlate with the pEBV. The mean pEBV accuracy (r^2^) for animals used to determine the gEBV accuracy is in Table 3. The validation subset was then further divided into categories based on genotyping of relatives in the training set. Validation animals were identified as having at least one parent (P), a least one parent and at least one offspring (PO), at least one offspring (O), or no parent and no offspring (N) genotyped in the training set. Accuracies of gEBV were then evaluated based on these categories, which is similar in principle to Habier *et al.* (2010). Additionally, genomic relationship coefficients were calculated between training and validation animals [using VanRaden (2008)], and the number of coefficients exceeding a threshold of 0.45 in each validation animal was determined, indicating the number of animals with which an individual was considered highly related. This value was then used as an additional categorization of the gEBV accuracy to determine the impact of knowing the actual relationship between the validation and training sets *vs.* knowing only the average relationship from the pedigree. The results of the 6-fold cross-validation were then compared with an approach where young animals with no progeny in the data compose the validation set (N = 509) and their parents (and other older animals) are in the training set (YoungVal). This approach is commonly used to validate gEBV in other livestock species in which progeny-tested pEBV are routinely available (*e.g.* Hayes *et al.* 2009; VanRaden *et al.* 2009). Accuracies of gEBV were estimated using the animals with the highest pEBV accuracies for each trait (N = 30). Because of very low pEBV accuracies (Table 3), Traits 1 and 2 were not included.

**Table 3:** Mean accuracy (r^2^) of estimated breeding values for individuals in the validation sets of the 6-fold cross-validation (XVal) and the validation using young animals (YoungVal), for all traits

	XVal		YoungVal	
Trait	Mean	SD	Mean	SD
T1	0.753	0.104	0.420	0.029
T2	0.813	0.055	0.590	0.032
T3	0.920	0.023	0.858	0.049
T4	0.945	0.016	0.906	0.032
T5	0.951	0.013	0.914	0.030

## Results

### Comparison of methods

**Figure 2:**
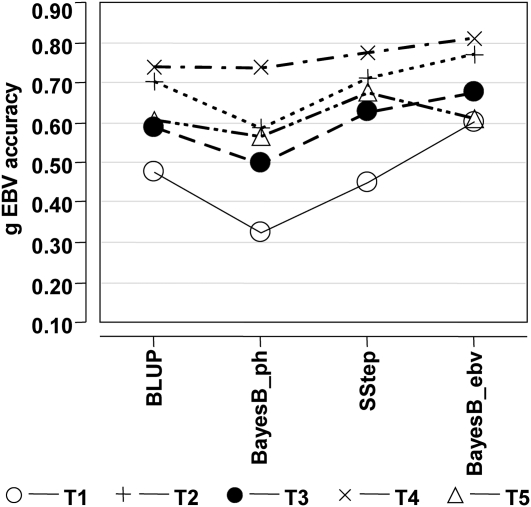
Genomic breeding value accuracy of the 6-fold cross-validation (XVal), for all traits, using a standard polygenic BLUP (BLUP), BayesB with phenotypes (BayesB_ph), the single-step approach (SStep), and BayesB with estimated breeding values (BayesB_ebv).

The gEBV accuracy generally increased with increasing heritability of the trait (Figure 2 and Table 1), corresponding to an increase in the mean pEBV accuracy for the validation samples (Table 3). The maximum accuracy was observed for trait (T)4 and the minimum for T1, across all methods (Figure 2). All correlations (between gEBV and pEBV) were different than zero (results not shown), but the low mean pEBV accuracy, especially for T1, suggests that the gEBV accuracy for this trait may be somewhat underestimated. The gEBV accuracy for T2 was nearly as high as the accuracy for T4, which was unexpected due to the lower mean EBV accuracy and the lower heritability of T2.

Across methods, the gEBV accuracy generally increased as additional information was implicitly utilized by the different approaches. Accuracies for all traits improved using SStep, compared with BayesB_ph, and improved again using BayesB_ebv, except for T5, in which there was a small decrease. The BayesB_ebv approach used information from progeny and a multigenerational pedigree to generate a higher accuracy “phenotype” (in the form of a de-regressed EBV), whereas the SStep approach used phenotypes much less representative of the additive value but included genomic relationships. The difference in accuracy between the two methods was generally small, with the exception of T1. The difference between methods differed across traits, with only small changes when the accuracy was higher but much larger changes with lower starting accuracies (and trait heritability). The addition of genomic data was expected to increase accuracy in all cases, compared with BLUP, but the phenotype-based BayesB_ph analysis resulted in accuracies that were actually lower (or nearly the same) than the standard analysis using pedigree alone.

### Relatedness between training and validation

**Figure 3:**
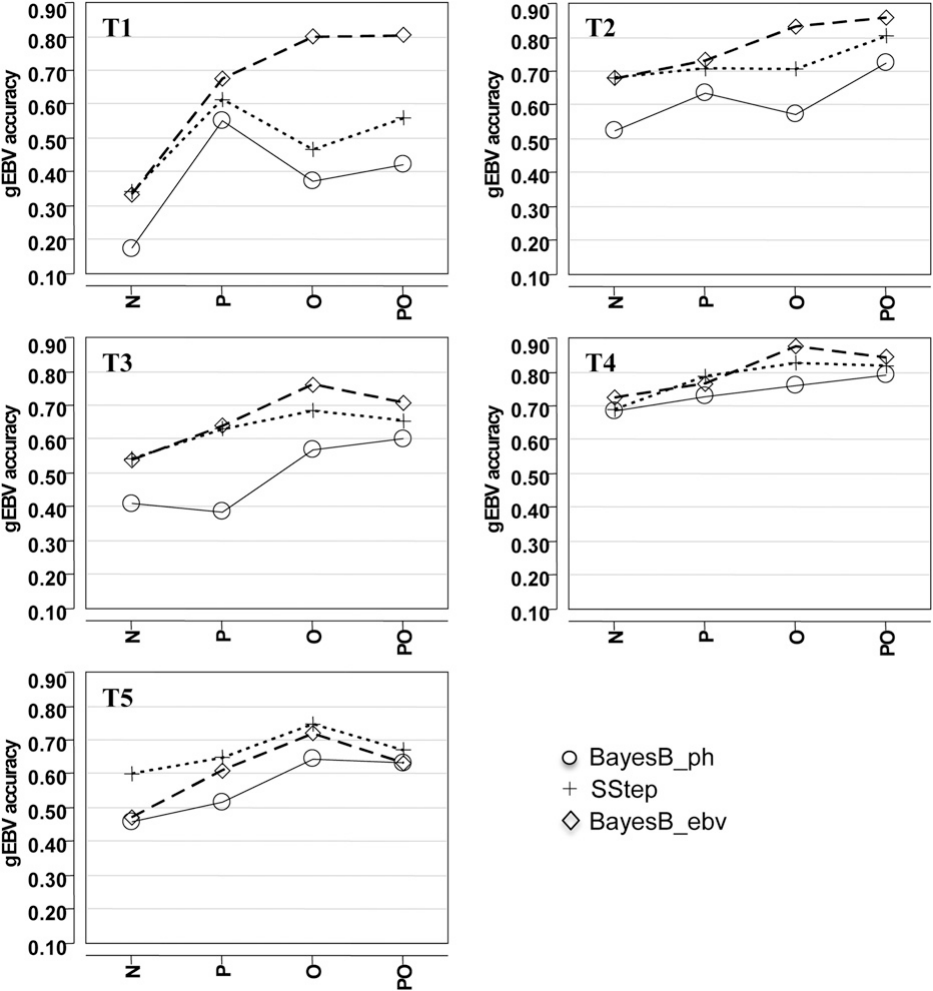
Genomic breeding value accuracy of the 6-fold cross-validation (XVal) for individuals with at least one parent (P), at least one offspring (O), at least one parent and one offspring (PO), or no parents or offspring (N) genotyped in the training set. All traits were analyzed with BayesB with phenotypes (BayesB_ph), the single-step approach (SStep), and BayesB with estimated breeding values (BayesB_ebv).

**Figure 4:**
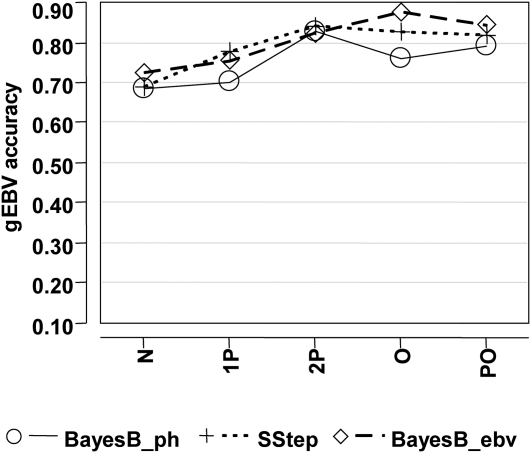
Genomic breeding value accuracy of the 6-fold cross-validation (XVal) for individuals with one parent (1P), both parents (2P), at least one offspring (O), at least one parent and one offspring (PO), or no parents or offspring (N) genotyped in the training set, for T4. The trait was analyzed with BayesB with phenotypes (BayesB_ph), the single-step approach (SStep), and BayesB with estimated breeding values (BayesB_ebv).

The gEBV accuracies increased with an increase in the relatedness between the two datasets (Figure 3). The change in accuracy with higher relatedness tended to be smaller as the trait heritability increased, as did the differences between methods. Very high gEBV accuracies were evident for some traits (*e.g.* T1) when including offspring in the training set, where accuracies approached 0.90. A gEBV accuracy of this magnitude is substantially larger than what would generally be expected, especially for a lowly heritable trait. An additional separation of accuracy by number of parents genotyped in training for T4 (Figure 4) shows that much of the accuracy increase is being driven by having both parents genotyped in the training population, which can be an advantage for populations that routinely genotype both sires and dams.

**Figure 5:**
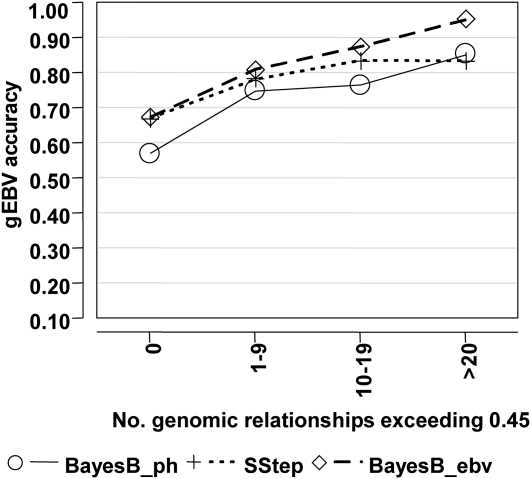
Genomic breeding value accuracy of the 6-fold cross-validation (XVal) for bins representing the number of validation animals having genomic relationship coefficients with animals in training that exceed 0.45, for T4. The trait was analyzed with BayesB with phenotypes (BayesB_ph), the single-step approach (SStep), and BayesB with estimated breeding values (BayesB_ebv).

The evaluation of relatedness using an index of the genomic relationship coefficients showed an increase in the gEBV accuracy for T4 for all methods as the magnitude of the genomic relationship increased (Figure 5). The other traits showed the same general trend but were slightly less consistent (results not shown). The minimum accuracies were lower and maximums higher when evaluating actual genomic relationships compared with the same approach using pedigree relationships (*e.g.* Figure 4), indicating that a more precise definition of the relationship between training and validation can impact the estimation of expected gEBV accuracy.

**Figure 6:**
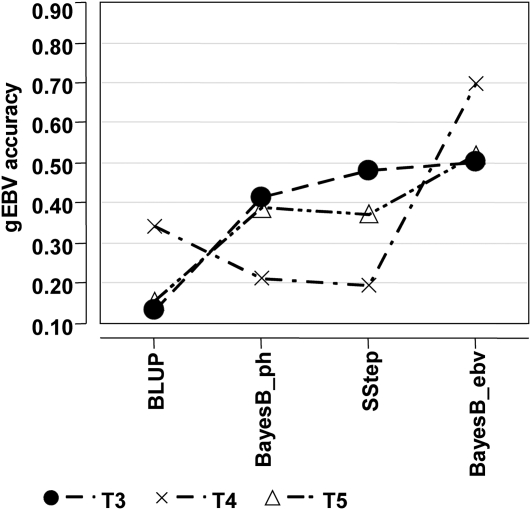
Genomic breeding value accuracy of the validation set containing young animals (YoungVal), for all traits, using a standard polygenic BLUP (BLUP), BayesB with phenotypes (BayesB_ph), the single-step approach (SStep), and BayesB with estimated breeding values (BayesB_ebv).

In the validation using young animals (YoungVal), there was an increase in gEBV accuracy moving from BLUP to BayesB_ebv (Figure 6), similar to the trend observed in XVal (Figure 2), although the correlations (accuracies) for BLUP were not different than zero (*P* > 0.05) in this case. The ranking of traits for gEBV accuracy was equivalent to the XVal for BLUP and BayesB_ebv, but reversed for BayesB_ph and SStep. Overall, the gEBV accuracies were shifted downward compared with XVal, likely due to a combination of lower accuracy pEBV and a reduction in the level of relatedness between training and validation sets. The gEBV accuracy for BayesB_ebv on T4 was similar to the accuracy obtained from the (N) and (P) categories in XVal (Figure 3), which was expected based on the relationships between the datasets in this validation, but the gEBV accuracies using BayesB_ph and SStep were much lower (∼0.20 compared with ∼0.70). The accuracies for T3 and T5 were more similar, but smaller than the cross-validation (XVal).

## Discussion

A dataset was created and a strategy developed to evaluate the accuracy of genomic selection approaches in livestock populations, especially when targeting populations where progeny-tested breeding values are not available on large numbers of individuals. The dataset was established for the purpose of implementing genomic selection in a production environment and so research utilizing the data can have direct implications for the application of genomic tools in breeding programs. These, and similar, data have been extensively analyzed within PIC and have been used to show the potential value of genomics in pig breeding, in terms of increased EBV accuracy at an early age (Cleveland *et al.* 2010; Forni *et al.* 2010; Deeb *et al.* 2011), to describe the genetic architecture of a commercial pig population (Deeb *et al.* 2010), and to develop alternative methodologies for genomic prediction (Forni *et al.* 2011; González-Recio and Forni 2011). The structure of this dataset allows for testing approaches considering varying levels of pedigree and/or genomic relationships because animals from multiple generations and both sexes have high-density genotypes. The structure also highlights the need to develop methods to account for a distribution of EBV accuracies when validating the accuracy of gEBV. Genus plc encourages researchers who wish to explore this (and other) datasets further to discuss potential collaborations.

The dataset enabled the testing of popular genomic prediction methods using real data, while the availability of both phenotypes and progeny-tested pEBV allowed for inferences about the value of sources of phenotypic information to different methods. The BayesB approach using de-regressed pEBV (BayesB_ebv) outperformed the other methods across almost all traits, which was not unexpected due to the nature of the data. Because they result from a production genetic evaluation using all available data, the pEBV contain information on the individual and its relatives in the full PIC pedigree. The SStep approach yielded accuracies similar to BayesB_ebv, but smaller in most cases. No strong evidence exists to support the existence of major QTL for these traits, and therefore, BayesB would not be expected to have a large advantage (results not shown). For traits affected by large numbers of loci (genes), the expectation is that a genomic BLUP approach should perform similarly to BayesB (Luan *et al.* 2009; Habier *et al.* 2010). In SStep, however, genomic relationships are augmented by pedigree relationships between genotyped and un-genotyped animals for a more accurate modeling of individual relationships, especially when only a subset of the population is genotyped (Legarra *et al.* 2009). Assuming no large QTL exist, any large differences between BayesB_ebv and SStep may be due to differences in the information content of the phenotypes used by each method and to differences in the training set size relative to differences in effective heritability. SStep outperformed BLUP, indicating that genomic information was useful, but only when the heritability was high. These findings, taken together, highlight the need for additional genotyping to improve lowly heritable traits using genomics, particularly when only phenotypes are available.

Including genomic information in a genetic evaluation is expected to increase the accuracy of the EBV, but the results presented here (Figure 2) illustrate the potential to actually decrease the accuracy when analyzing phenotypes, especially when the heritability is low. When heritability is not high, large numbers of genotyped and phenotyped animals are needed to achieve even moderate gEBV accuracy (Goddard 2009; Goddard and Hayes 2009). Such numbers were not available in this dataset. Alternatively, the heritability of the trait can be increased by using de-regressed progeny test EBV as phenotypes, in which the effective heritability is proportional to the pEBV accuracy (Garrick *et al.* 2009). The expected accuracy of the gEBV will then be based on this higher heritability (*e.g.* BayesB_ph *vs.* BayesB_ebv). Often, the number of available genotyped samples is based on practical or budgetary considerations, and the decision to incorporate genomic information for a particular trait and the method that will be used depends on the heritability and the availability of progeny and pedigree information.

The gEBV accuracy based on relatedness between training and validation sets increased with an increase in pedigree relationship (Figures 3 and 4). Using a count of genomic coefficients exceeding a threshold showed this relationship even more clearly (Figure 5). These results indicate that expected gEBV accuracies from validation could be biased when highly related individuals are in both training and validation. This relatedness may cause an overestimation of expected accuracies when the target application consists of less-related samples (Habier *et al.* 2010). In practice, the population used for training is often composed of the parents and ancestors of the individuals for which gEBV are required. A certain amount of relatedness between populations is appropriate, but the validation should aim to simulate the application as closely as possible. Interpretation of the accuracy of gEBV without knowledge of the pedigree relationships within the population is not optimal.

For pig populations, where genomic predictions of young selection candidates within a single line are often desired, creating a validation dataset using young animals with high accuracy pEBV is difficult due to the structure of the breeding program. Because relatively few individuals have large amounts of progeny information, it is generally impossible to high-density genotype large numbers of individuals with high-accuracy pEBV. The younger animals in the dataset that may be used in validation are typically not suitable because of low-accuracy pEBV for many traits, which would provide a poor estimate of gEBV accuracy. Using older animals in this role would require removing their progeny from the training set, reducing the training set size to unacceptable levels. The small test performed here resulted in gEBV accuracies from YoungVal (Figure 6) that were smaller than those in XVal (Figure 2), but when accuracies were evaluated in XVal considering pedigree and genomic relationships, they were similar to those from YoungVal. These results suggest that when it is not possible to simulate the application of a genomic evaluation for validation purposes, such as for T1 and T2 in this study, a k-fold cross-validation can provide appropriate estimates of gEBV accuracy when specifying the relatedness between training and validation. Additionally, genomic relationships may help determine the bounds of potential gEBV accuracy given the projected relatedness between datasets.

### Implications

The results of this study indicate that the differences between methods to predict gEBV depend primarily on the information content of the phenotype, rather than on the relationship between training and validation, as expressed by the minimal level of reranking between methods. The conclusions drawn concerning the impact of relatedness on expected accuracies from validation can also be applied to genotyping strategies for implementing genomic selection programs. The relatedness between the training and prediction populations will influence the gEBV accuracy, and therefore, high levels of relatedness are desirable for breeding program design. Such high levels are most easily accomplished by genotyping both parents of prediction animals, but more selective genotyping may be possible by maximizing genomic relationships between the populations in which more distant pedigree relationships may be useful.

## Supplementary Material

Supporting Information
