# Supplementary material for: A Common Dataset for Genomic Analysis of Livestock Populations
Source: G3 (Bethesda). 2012 Apr 1;2(4):429–35. doi: 10.1534/g3.111.001453 (PMC3337471; doi:10.1534/g3.111.001453)
Supplement: Supporting Information [file supp_2_4_429__index.html]

Supporting Information 

# A Common Dataset for Genomic Analysis of Livestock Populations

## Supporting Information for Cleveland, Hickey, and Forni, 2012

**Files in this Data Supplement:**

- File S1 - PIC Data (.zip, 67.5 MB)
