# Supplementary material for: A Common Dataset for Genomic Analysis of Livestock Populations
Source: G3 (Bethesda). 2012 Apr 1;2(4):429–35. doi: 10.1534/g3.111.001453 (PMC3337471; doi:10.1534/g3.111.001453)
Supplement: Supporting Information [file supp_2.4.429_FileS1.zip › FileS1/PIC Data Description.docx]

**PIC Data: Description of Files**

All files are comma-separated, with a header

pedigree.txt: File listing pedigree for the genotyped animals, includes all genotyped animals plus all sires, dams, grand-sires and grand-dams (where known). Unknown parents are denoted by “0”.

phenotypes.txt Phenotypes for all genotyped animals, for 5 traits. Phenotypes are corrected for fixed effects or are weighted progeny mean corrected phenotypes. Missing phenotypes are denoted by “.”. Heritability for each trait is: t1=0.07, t2=0.16, t3=0.38, t4=0.58, t4=0.62.

ebvs.txt Estimated breeding values for all genotyped animals, for all 5 traits, plus accuracies (reliabilities) for each EBV. The EBV come from the full PIC genetic evaluation. There are no missing values.

genotypes.txt Genotypes for all genotyped individuals for 52,843 SNPs (after removing monomorphic and low-coverage SNPs, and sex chromosomes). Missing genotypes were imputed, so the resulting genotypes may be non-integer, ranging from 0 to 2 (called genotypes are integers 0,1,2). The SNP order was randomized and the SNP identities assigned sequentially. There are no missing values.
